# Acid Sensing Ion Channel 1 in Lateral Hypothalamus Contributes to Breathing Control

**DOI:** 10.1371/journal.pone.0039982

**Published:** 2012-07-06

**Authors:** Nana Song, Guihong Zhang, Wenye Geng, Zibing Liu, Weizhong Jin, Li Li, Yinxiang Cao, Danian Zhu, Jerry Yu, Linlin Shen

**Affiliations:** 1 Department of Physiology and Pathophysiology, Shanghai Medical College, Fudan University, Shanghai, China; 2 Department of Pulmonary Medicine, University of Louisville, Louisville, Kentucky, United States of America; Virginia Commonwealth University, United States of America

## Abstract

Acid-sensing ion channels (ASICs) are present in neurons and may contribute to chemoreception. Among six subunits of ASICs, ASIC1 is mainly expressed in the central nervous system. Recently, multiple sites in the brain including the lateral hypothalamus (LH) have been found to be sensitive to extracellular acidification. Since LH contains orexin neurons and innervates the medulla respiratory center, we hypothesize that ASIC1 is expressed on the orexin neuron and contributes to acid-induced increase in respiratory drive. To test this hypothesis, we used double immunofluorescence to determine whether ASIC1 is expressed on orexin neurons in the LH, and assessed integrated phrenic nerve discharge (iPND) in intact rats in response to acidification of the LH. We found that ASIC1 was co-localized with orexinA in the LH. Microinjection of acidified artificial cerebrospinal fluid increased the amplitude of iPND by 70% (pH 7.4 *v.s.* pH 6.5∶1.05±0.12 *v.s.* 1.70±0.10, n = 6, P<0.001) and increased the respiratory drive (peak amplitude of iPND/inspiratory time, PA/Ti) by 40% (1.10±0.23 *v.s.* 1.50±0.38, P<0.05). This stimulatory effect was abolished by blocking ASIC1 with a nonselective inhibitor (amiloride 10 mM), a selective inhibitor (PcTX1, 10 nM) or by damaging orexin neurons in the LH. Current results support our hypothesis that the orexin neuron in the LH can exert an excitation on respiration via ASIC1 during local acidosis. Since central acidification is involved in breathing dysfunction in a variety of pulmonary diseases, understanding its underlying mechanism may improve patient management.

## Introduction

Acid-sensing ion channels represent an H^+^-gated subgroup of the amiloride-sensitive Na^+^ channel/degenerin family (ENaC/DEG), a family of cation channels [1]. Six subunits have been identified: ASIC1a, ASIC1b, ASIC2a, ASIC2b, ASIC3 and ASIC4 [2]. Both homomeric and heteromeric ASICs tetramers can be formed with different kinetics, pH sensitivities (ASIC1a: pH_0.5_ = 6.5, ASIC1b: pH_0.5_ = 5.9, ASIC2a: pH_0.5_ = 4.7) [3], [4], and tissue distributions [5]–[7]. ASIC2b and ASIC4 subunits can only operate in the form of heteromers with other subunits [5], [8]. ASICs are widely expressed in peripheral and central nervous systems (CNS) and involved in physiological and pathophysiological functions, such as sour taste [9], hearing [10], [11], and cutaneous/visceral mechanosensation [12]–[15]. In the CNS, neurons express ASIC1a, ASIC2a, ASIC2b and ASIC4 subunits [16], but predominantly ASIC1a. ASIC1a have been identified in brain regions, including the glomerulus of the olfactory bulb, striatum, nucleus accumbens, amygdale, and hippocampus, and whiskey barrel, cingulate, and cerebellar cortexes [17], [18]. ASIC1a modulates synaptic plasticity, contributes to learning and memory, and is important in fear related behavior [17]. Most early work on central chemoreceptors focused on the brainstem. In the 1950s, Redgate and Gellhorn found that injection of barbiturates into the LH reduced respiratory activity [19]. These studies established that the LH may exert an excitatory drive to respiration. Recent data revealed orexin containing neurons located in the LH were related to control of breathing and arousal [20], [21]. Orexin cell in vitro can be potently stimulated by CO_2_ and H^+^ [22]. It seems possible that the LH may monitor the brain acidity in vivo. In the present studies, we hypothesize that ASIC1a located on the orexin neurons in the LH contribute to the regulation of breathing by sensing local acidity. To test the hypotheses, we performed immunohistochemical staining to examine whether ASIC1 co-express with orexinA. Since the effect of acidification of the LH on respiration has never been reported in intact animals, we also examined phrenic nerve activity in response to LH acidification with or without blocking ASIC1a. Our data support that acidification of the LH can stimulate breathing via activation of ASIC1a on orexin neurons.

**Figure 1:**
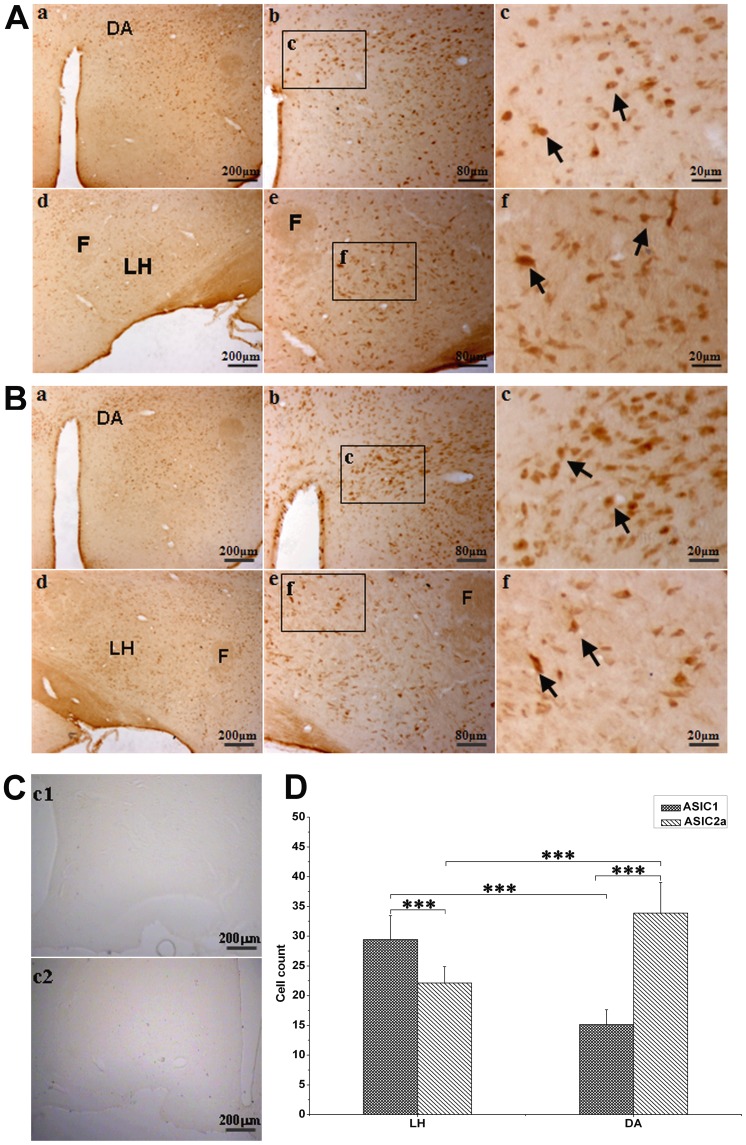
Distribution of ASIC1-ir and ASIC2a-ir neurons in the hypothalamus. A: ASIC1-ir neurons in the dorsal hypothalamus area (DA) (a, b, c) and in the lateral hypothalamus (LH) (d, e, f). B: ASIC2a-ir neurons in the DA (a, b, c) and the LH (d, e, f). C: c1∶1% BSA controls; c2: The peptides absorbed antibody control. D: Group data show the numbers of ASIC1- and ASIC2a-positive cells per visual field under microscope (×200) in the LH and DA. (*** P<0.001, n = 6).

## Results

### 1. Expression of ASIC1 and ASIC2a in Hypothalamus

**Figure 2:**
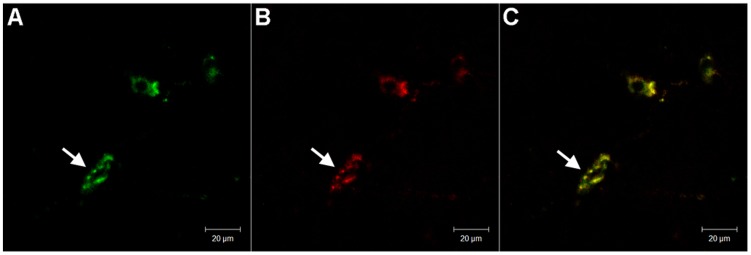
Co-expression of ASIC1 and OrexinA in the LH of adult SD rats. A representative confocal photomicrograph showing co-staining of orexinA and ASIC1 on neurons in the LH of an adult SD rat. **A:** OrexinA-ir neurons (green, FITC). **B:** ASIC1-ir neurons (red, cy3). **C:** Overlay of A and B.

Both ASIC1-ir (immunoreactive) (Fig. 1A) and ASIC2a-ir (Fig. 1B) neurons were expressed in the hypothalamus. They were concentrated in the LH and dorsal hypothalamus area (DA), but with different distributions. In the LH, ASIC1-ir cells [29.4±4.0 count/visual field (C/VF)] were more populous than ASIC2a-ir cells (22.1±2.7 C/VF, n = 7), P<0.001, whereas in the DA, it was vice versa (15.1±2.5 C/VF for ASIC1-ir vs 33.9±5.1 C/VF for ASIC2a-ir, P<0.001, n = 7, Fig. 1D). Clearly, ASIC1-ir neurons were more in the LH than in the DA (P<0.001). In the LH, some neurons were co-stained with ASIC1 and OrexinA (Fig. 2).

### 2. Effect of Microinjection of Acidic ACSF to LH on PND

**Figure 3:**
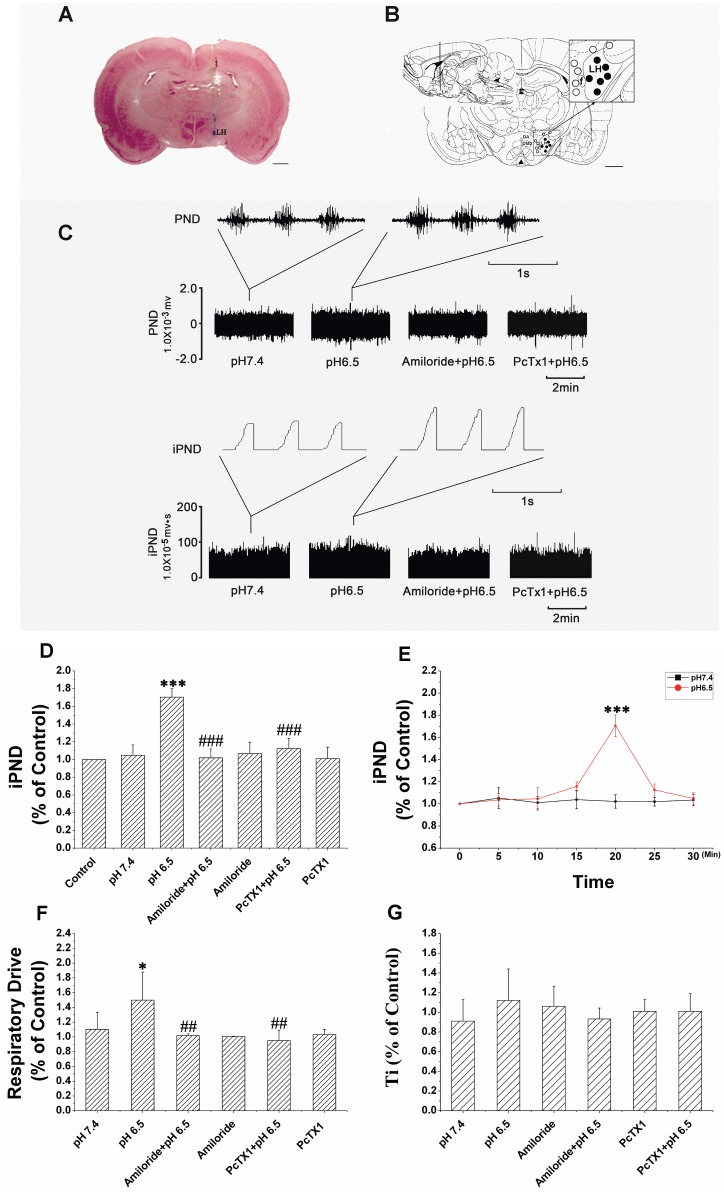
ASICs antagonist blocking acidification-induced increase of PND. A: Histological staining with neutral red: the sky blue spot was the injection site in the LH. Scale bar, 1 mm. B: Plot of the injection sites projected on Bregma −2.6 mm section: the solid dots were inside and the hollow ones outside of the LH. Scale bar, 1 mm. C: Unilateral microinjection of 0.1 µl ACSF (pH 6.5) into the LH increased raw PND (top) and iPND (bottom). Pre-treatment of amiloride (10 mM) or PcTX1 (10 nM) blocked the effect. Microinjection of ACSF (pH 7.4) served as the control. D: Group data show the effects of amiloride and PcTX1 on acidification-induced iPND (n = 6, *** P<0.001 relative to control, ^###^ P<0.001 relative to pH 6.5). E: Time course of iPND response to acidification of the LH. Please note that the response peaked at 20 min (n = 6, *** P<0.001 relative to pH 7.4). F: Responses of respiratory drive (PA/Ti). Note that amiloride and PcTX1 inhibited the acidification-induced effect (n = 6, ^#^ P<0.05 relative to pH 7.4, ** P<0.01 relative to pH 6.5). G: Inspiratory time (Ti) was increased by acidification but not statistically significant (*v.s.* pH 7.4).

Acidifying the LH (with ACSF from pH 7.4 to 6.5) stimulated respiration. The stimulation became apparent at 15 min following acidification, reaching a peak at 20 min. At the peak response, iPND increased by approximately 70% (from 1.05±0.12 to 1.70±0.10, n = 6, P<0.001, Figs. 3D and 3E). The respiratory drive (PA/Ti) also increased by about 40% (from 1.10±0.23 to 1.50±0.38, n = 6, P<0.05, Fig. 3F). The stimulatory effect lasted about 4 min. Inspiratory time (Ti) was prolonged, but was not statistically significant (0.99±0.22 *v.s.* 1.12±0.32, Fig. 3G). Acidification had no effects on mean arterial pressure (MAP), heart rate (HR) and respiratory rate (RR) (Table 1). Microinjection points were verified histologically (Figs. 3A and 3B). Acidification outside of the LH had no effect on PND.

**Table 1:** Effect of microinjection of ACSF with different pH and ASICs inhibitor into LH on RR, MAP and HR.

	RR(bpm)	MAP(mmHg)	HR(bpm)
Control	108.70±23.23	77.55±11.50	437.03±42.82
pH 7.4	114.28±20.27	74.75±8.27	424.47±63.81
pH 6.5	124.69±27.94	77.34±7.36	424.78±52.03
Amiloride+pH 6.5	118.17±31.05	83.23±13.38	428.11±77.29
Amiloride	99.55±20.74	58.03±13.30	434.03±45.49
PcTX1+pH 6.5	106.00±10.18	78.47±19.89	425.61±31.28
PcTX1	104.17±5.46	79.92±22.05	418.59±45.32

### 3. ASICs Antagonist Blocking Effect of Acidic ACSF on PND

Pre-treatment with a nonselective ASICs inhibitor (amiloride, 10 mM) or a selective ASIC1a inhibitor (PcTX1, 10 nM) did not alter iPND and respiratory drive during resting conditions, but almost blocked the increases in iPND and respiratory drive (Figs. 3D and 3F) induced by microinjection of acidic ACSF into the LH (both n = 6 and P>0.05).

### 4. Damage of orexinA Neurons Blocking Effect of Acidification

**Figure 4:**
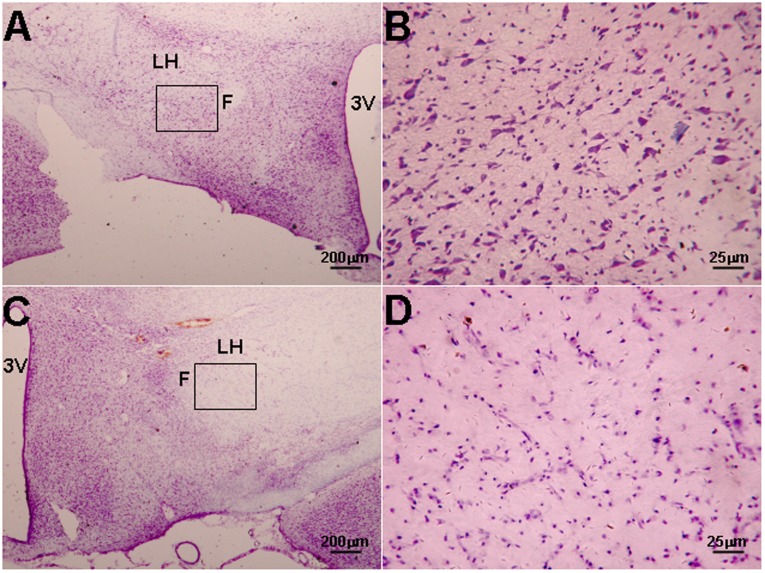
Loss of Nissl bodies in the LH after orexin-SAP treatment. A: Nissl's staining of coronal section in the blank-SAP-treated rat. B: Higher magnification of the square area in A. C and D are the same as A and B but from the orexin-SAP treated rat. There was a significant loss of Nissl bodies after the LH lesion caused by orexin-SAP-treatment. 3V: third ventricle, LH: lateral hypothalamus, F: Fornix.

**Figure 5:**
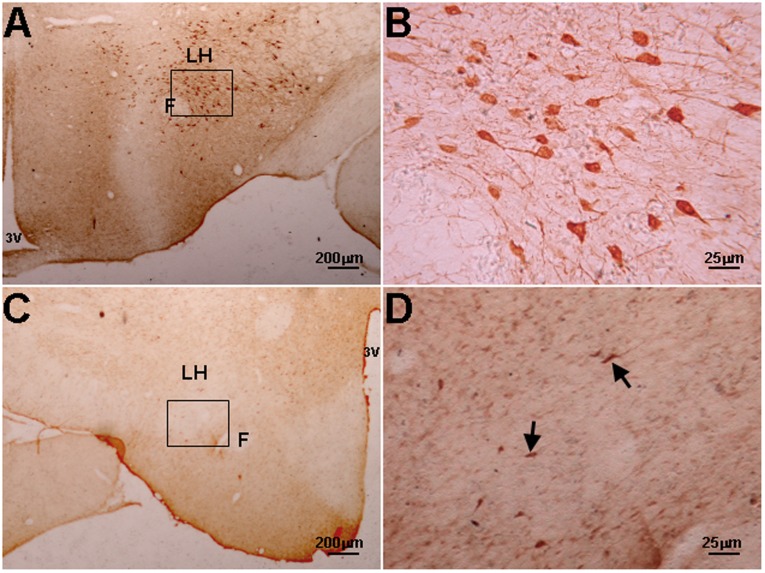
Loss of orexinA-immunoreactive neurons in the LH after orexin-SAP treatment. A and B are low and high magnification photomicrographs of orexin-ir neurons in coronal sections of the brain in the blank-SAP-treated rat. C and D as A and B, but from the orexin-SAP-treated rat. Orexin neurons were significantly fewer after damage of the LH by orexin-SAP-treatment with a few residual ones indicated by black arrows. 3V: third ventricle, LH: lateral hypothalamus, F: Fornix.

**Figure 6:**
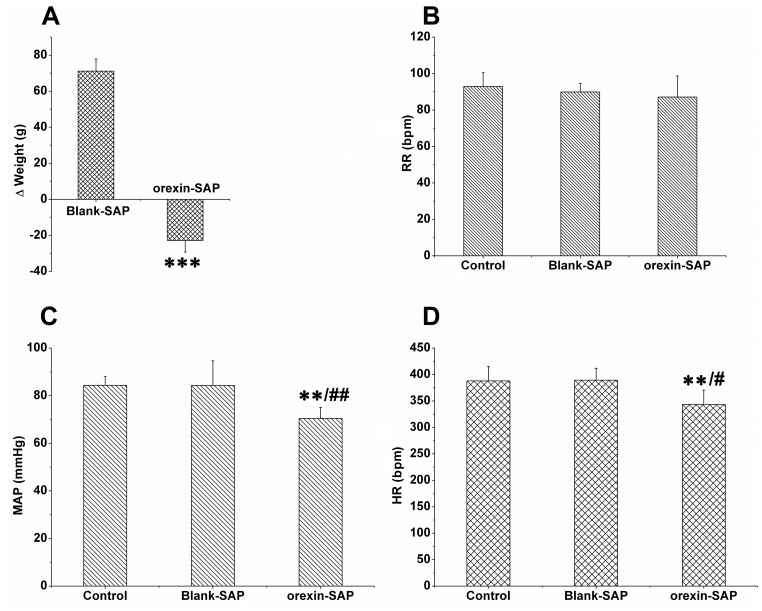
Effect of the LH lesion on body weight, RR, MAP and HR. Two weeks after microinjection of orexin-SAP into the LH, A: there was a weight loss (A, ***p<0.001, n = 6, compared with blank-SAP treated group) with no change in respiratory rate (B, RR). However, mean arterial pressure (MAP, C) and heart rate (HR, D) decreased (**p<0.01 v.s. blank-SAP treated group, ^##^ p<0.01 *v.s.* control,^ #^ p<0.05 *v.s.* control).

Two weeks after treatment of the LH with orexin-SAP, there was a significant loss of Nissl bodies with few residual orexin neurons remaining (Figs. 4 and 5). After the lesions, body weight, MAP and HR decreased with no change in respiration (Fig. 6). Microinjection of acidic ACSF into the LH no longer stimulated breathing (Table 2).

**Table 2:** Effect of microinjection of ACSF with different pH on breathing in rats with damaging of the LH.

	Orexin-SAP		Blank-SAP		
	iPND	RR (bpm)	iPND	RR (bpm)	
Control	1.00±0.00	87.14±11.51	1.00±0.00	89.89±4.61	
pH7.4	0.99±0.14	85.32±10.24	1.02±0.19	88.51±10.33	
pH6.5	1.08±0.21	87.43±9.46	1.62±0.25**	89.26±14.27	

** p<0.01, n = 6.

## Discussion

Our current data demonstrated that ASIC1a expressed on the orexin neurons in the LH contributed to the regulation of breathing. Since 1997, when Waldmann cloned a subunit of H^+^-gated channels (ASICs) transiently activated by rapid extracellular acidification [1], ASICs have been considered for chemoreception. ASICs are widely expressed in the CNS with ASIC1 and ASIC2a being the most prevalent [18], [23], [24]. Similarly, we found that the ASIC1-ir and ASIC2a-ir neurons throughout the hypothalamus with different densities in different regions. ASIC1-ir neurons are mainly located in the LH (Fig.1A, 1D). Different subunits of ASICs have different pH sensitivities, which determine their physiological function. Homomeric ASIC1a channels have a half-maximal activation pH (pH_0.5_) between 6.2 and 6.8 [25], [26], therefore, pH of 6.5 was used to acidify the LH in the present studies. Acidification to this pH stimulated breathing, supporting that ASIC1a channels were responsible (Fig.3). Although ASIC2a also expressed in the hypothalamus, it is relatively insensitive to H^+^. ASIC2a homomultimers have a very low pH threshold for activation (∼pH 4.8) with a pH_0.5_ of 4.4. Such a low pH sensing range is unlikely to be responsible for detecting physiological changes in extracellular pH. At this moment, we do not know the physiological function of ASIC2. However, ASIC2a may modulate ASIC1 H^+^-activated currents [27]. Conventionally, ASIC3 is believed to be peripherally located, and does not express in the CNS [7], [23]. However, recently, ASIC3 has been detected in the neurons of hypothalamus [28], and other brain areas (along with ASIC1b, ASIC2a) that are critical for central chemoreception, such as trapezoid body and lateral paragigantocellular nucleus [29]. These ASICs may participate in chemoregulation of breathing. Indeed, ASIC3 has a high sensitivity to protons, with a pH_0.5_ close to 6.5. Thus, ASIC3 may also contribute to increased breathing in our current studies. However, ASIC currents in the hypothalamic neurons are characterized by fast desensitization and pH_0.5_ values consistent with ASIC1a properties [30]. In addition, selective blockade of ASIC1a with PcTX1 abolished the acid induced effect, which suggests that ASIC1a is responsible for acid sensing in the hypothalamus and ASIC3 if had any effect will be minor in our studies.

Since ASIC1 responds to acidification with rapid desensitization in seconds, which seems contradict with the sustained effect on breathing. However, ASIC1a is unique. In its homo-multimeric form, it is not only permeable to Na^+^ but also to Ca^2+^ [31], [32]. During persistent acidosis, although ASIC1a is desensitized within a few seconds [33], intracellular Ca^2+^ concentration increases gradually with a long time constant in the order of minutes [34]. Increased intracellular Ca^2+^ is known to mediate numerous neuronal activities. Thus, while we do not know why acidification activates ASIC1a with a rapid desensitization and a sustained stimulatory effect on breathing, the observation is not surprising. For example, acid-stimulated duodenal mucosal bicarbonate secretion in vivo was ASIC1a mediated via a Ca^2+^ signaling pathway. The secretion effect was long lasting, which peaked about 25 minutes following the acidification [35].

The breathing of mammals is controlled by a neuronal network in response to peripheral and central inputs, especially from chemical stimuli, such as CO_2_ and H^+^. The hypothalamus is an integrating center for the cardiopulmonary system and is involved in this network. Lateral hypothalamus lesions result in immediate inhibition of breathing [19]. Microinjections of D,L-homocysteic acid into the hypothalamus also increase phrenic activities *in vivo* [36]. CO_2_ inhalation increase Fos-like immunoreactivity in the hypothalamus [37], [38] and activate orexin neurons in the hypothalamus of the mouse [39]. Thus, the orexin system originating from the LH might contribute to respiratory regulation. Indeed, orexin neurons found in the LH can be stimulated by CO_2_/H^+^ [22], [39]. Breathing stimulation caused by increased CO_2_ or acidity in the LH is suppressed in the orexin knockout mice [40], [41]. Patch-clamp recordings in LH mouse brain slices show acidosis stimulates orexin cell firing while alkalosis inhibits [22]. In addition, orexin neurons in the LH project directly into respiration related sites, such as the Pre-Bötzinger complex, hypoglossal and phrenic nuclei, and nucleus tractus solitarius [42]–[44]. Microinjection of orexin A into the medulla stimulates breathing [44] and knockout of orexin attenuates hypercapnea-induced ventilatory responses [41]. Taken together, it seems that acidification of LH may stimulate orexin neuron via activation of ASIC1. Stimulation of orexin neurons may lead to orexinA release in the medulla respiratory center to modulate breathing pattern.

Researches have suggested that orexin neurons in the LH may contribute to central chemoreception [45], [46]. To be qualified as a central chemoreceptor, three criteria need to be met: (1) the sensory neuron should be activated by chemical stimuli (CO_2_/H^+^); (2) physiological intensity of the stimulus should increase ventilation; and (3) destruction or inhibition of the neuron should attenuate the hypercapnea-induced ventilatory augmentation [45]. While our present data support that orexin neurons in the LH can sense an increase in H^+^ that stimulates breathing, the underlying mechanisms differ from those operating in conventional central chemoreception. Conventional chemical reflexes regulate ventilation breath by breath and are essential in maintaining ventilation under physiological pH. The LH sensing system, however, detects a lower pH range (6.5) in local area. This precludes its role in physiological conditions, although the neuron can certainly be activated in a variety of pulmonary diseases with decreased pH. Indeed, blocking ASICs on the orexin neuron or destroying the neuron did not alter ventilation under resting conditions (Figs.3D, 3F andTab.2). Knockout of orexin or blocking orexin receptors does not alter resting ventilation, but attenuates the hypercapnic response during wakefulness [46]–[48]. Thus, the orexin neurons in the LH may provide a respiratory drive to the medulla respiratory center to control breathing during pathophysiological conditions, such as acidosis. In addition, lateral hypothalamus dysfunction involves several neurological and psychiatric disorders such as narcolepsy, anxiety, depression and mania and alters central CO_2_ chemoreception, where ASICs may play a role [49]–[51]. Understanding this mechanism of central chemoregulation will help management of patients with these diseases.

In summary, extracellular pH is a fundamental signal in the regulation of breathing. In the current studies, we have shown that ASIC1 is expressed in the LH and co-stained with orexinA on some neurons in the LH. These data support our hypothesis, however, they are not conclusive. More extensive research in this area is needed. Evidence from in situ hybridization is needed to support channel distribution, because conclusions based solely on immunochemical methods relies on the specificity of antibody recognition. Nevertheless, we have shown that local acidification in the LH can stimulate breathing, which is inhibited by selectively blocking ASIC1a or by damaging the orexin neurons in the LH. Our findings support that ASIC1 expressed on orexin neurons in the LH may participate in chemical regulation of breathing during acidosis.

## Materials and Methods

### 1. Animals

Experiments conformed to the Regulations for the Administration of Affairs Concerning Experimental Animals, National Committee of Science and Technology of China and Instructive Notions with Respect to Caring for Laboratory Animals, Ministry of Science and Technology of China, and were approved by the Ethics Committee for Experimental Research, Shanghai Medical College, Fudan University.

Male Sprague–Dawley rats (250–350 g) (Experimental Animal Center of Chinese Academy of Sciences in Shanghai) were anesthetized with a mixture of urethane (0.7 g kg^−1^), α-chloralose (35 mg kg^−1^) and borax (35 mg kg^−1^) intraperitoneally. Supplements (about tenth of the initial dose) were given hourly. Adequacy of anaesthesia was assured by absence of pedal withdrawal response. The femoral artery and external jugular vein were cannulated for arterial pressure and administration of fluids and drugs, respectively. Endotracheal intubation was also performed and animals were allowed to breathe spontaneously [52], [53]. Mean arterial pressure (MAP) and heart rate (HR) were calculated from the arterial pressure wave. The phrenic nerve was recorded by a dorsolateral approach. Rectal temperature was monitored and maintained at 37°C by a temperature controller (Quanshui H-KWDY-III), and fluid replacement was given by a syringe pump (HARVARD APPARATUS 11Plus) at the rate of 1mL/h.

### 2. Immunohistochemistry

#### 2.1. Tissue preparation

Seven rats were anesthetized, sacrificed, and perfused through the left ventricle with normal saline followed by 4% paraformaldehyde in 0.1 M phosphate buffer, pH 7.4. After perfusion, brains were removed and post-fixed by immersion in the same fixative overnight. The hypothalamus tissues were dissected, equilibrated in graded sucrose solution (20%, 30%) and coronally sectioned at 30 µm in one to five serial orders on Leica freezing microtome.

#### 2.2. Immunohistochemistry ABC method

Slides were washed in 0.01 M PBS (pH 7.4), incubated with the first antibody (ASIC1 or ASIC2a, Santa Cruz Biotechnology, rabbit anti rat IgG, 1∶100) diluted in 1% bovine serum albumin (1% BSA) buffer for 24 hours in 4°C. 1% BSA or a solution in which the antibody was absorbed by relevant peptides was used as controls. After 3 washes in 0.01 M PBS, the slides were incubated in 1%BSA for 1hour to block background staining, and the reaction was detected with avidin-biotin-HRP complex (ABC) immuno detect kit (Sino-American Biotechnology Co.). Sections were transferred onto glasses, dried in open-air, mounted after dehydration, and then examined under a microscope. Positive stained cells were counted in six sections of the LH and DA from each animal by a software (ImageMeasure, Shanghai Medical College, Fudan University, China) in a blinded manner to the treatment.

#### 2.3. Double immunofluorescence technique

Slides were washed in 0.05M Tris–Saline Buffer, pH 7.6 containing 0.1% Triton X-100 (TBSTx). The slides were incubated with a mixture of the first antibody of ASIC1 (Santa Cruz Biotechnology, goat anti rat IgG, 1∶50) and orexinA (Sigma Aldrich, rabbit anti rat IgG, 1∶100) diluted in 1% BSA for 24 hours. All dilutions had been established by preliminary titration. After 3 washes in TBSTx, the slides were incubated in a 1% mixture serum of donkey and goat for 1hour to block background staining, and then the reactions were detected with a mixture of donkey anti-goat IgG conjugated with cy3 (Beyotime Institute of Biotechnology) and goat anti-rabbit IgG conjugated with FITC (Beyotime Institute of Biotechnology) diluted 1∶200 in 1% BSA for 1 hour in the dark. After 3 washes in TBSTx, the slides were air-dried, and mounted in antifading medium (Beyotime Institute of Biotechnology), and examined with confocal laser scanning microscope (Zeiss 510).

### 3. Phrenic Nerve Discharge Recording

Activities in the left phrenic nerve were recorded with platinum bipolar electrodes, pre-amplified and bandpass filtered (5KHz) by a Polygraph System (NIHON KOHDEN), digitized by Bio-electric signals processing system (SMUP-E, Shanghai Medical College, Fudan University), and then integrated and stored using MFLab-200 software (Shanghai medical college, Fudan University, China) for subsequent analysis. The experiments were started after stabilization of the phrenic activity (about 30 min). The iPND was obtained by a moving average of the phrenic signal. PA and Ti were averaged over 30 s. PA/Ti was calculated to assess the respiratory drive. Respiration rate (RR) was computed from the phrenic discharge.

### 4. Microinjections

Unilateral microinjection, at a volume of 0.1 µl, was carried out stereotaxically and sequentially with a 27-gauge stainless steel (internal cannula connected to a 1 µl microliter syringe). The injection position was verified by histology at the end of the experiment. Test agents, a nonselective ASICs inhibitor (amiloride 10 mM, Sigma Aldrich) or a selective ASIC1a inhibitor (tarantula venom PcTX1 10 nM, Peptides International), were freshly prepared in artificial cerebrospinal fluid (ACSF) immediately before administration. ACSF were prepared at different pH (7.4, 6.5). Co-microinjection was performed with the inhibitors first followed by the effective pH immediately. ACSF with pH 7.4 served as the vehicle and volume control with the composition (mM): NaCl 130, NaHCO_3_ 26, KCl 5, CaCl_2_ 2.6, MgSO_4_ 1.2, NaH_2_PO4 1.6, glucose 11 and sucrose 10. To avoid the confounding effects of drug interactions, each animal received only one pharmacological treatment. At the end of the experiment, 0.1 µl pontamine sky blue was injected into the injection point. Brains were removed, fixed, frozen, coronally sectioned (30µm), and stained with neutral red for histological verification.

### 5. Lateral Hypothalamic Lesion with Orexin-SAP

Six rats were bilaterally microinjected with orexin-SAP or Blank-SAP (0.43 ng/nl, 400 nl each side, Advanced Targeting Systems, San Diego, CA, USA) into the LH (2.6 mm posterior, 1.6 mm lateral, and 7.0 mm dorsal from bregma) [54]. The agents were delivered via a glass micropipette with a tip diameter of 20µm, coupled to a pressure injector (Picospritzer; General Valve, Fairfield, NJ). After injection, the pipette was left in place for 5 min and then withdrawn slowly. Animals were allowed 14 days for lesions of orexin neurons to develop and for recovery. Then, the phrenic response to microinjection of ACSF with pH 6.5 was examined.

### 6. Statistics

Stata software was used for statistical analysis. Data are reported as means ± standard error of the mean (Mean ± SEM). The student *t*-test and one-way parametric ANOVA followed by Bonferroni tests were used as appropriate. Significance was set at P<0.05.
